# Clinical frailty scale is useful in predicting return-to-home in patients admitted due to coronavirus disease

**DOI:** 10.1186/s12877-023-04133-4

**Published:** 2023-07-13

**Authors:** Koki Kawamura, Aiko Osawa, Masanori Tanimoto, Hitoshi Kagaya, Toshihiro Matsuura, Hidenori Arai

**Affiliations:** National Center for Geriatrics and Gerontology, 7-430 Morioka-cho, Obu, 474-8511 Aichi Japan

**Keywords:** COVID-19, Frailty, Predicts discharge, Epidemic

## Abstract

**Background:**

The spread of the novel severe acute respiratory syndrome coronavirus 2 infection has been prolonged, with the highly contagious Omicron variant becoming the predominant variant by 2022. Many patients admitted to dedicated coronavirus disease 2019 (COVID-19) wards (COVID-19 treatment units) develop disuse syndrome while being treated in the hospital, and their ability to perform activities of daily living declines, making it difficult for hospitals to discharge them. This study aimed to investigate the relationship between the degree of frailty and home discharge of patients admitted to a COVID-19 treatment units.

**Methods:**

This study retrospectively examined the in-patient medical records of 138 patients (82.7 ± 7.6 years old) admitted to a COVID-19 treatment unit from January to December 2022. The end-point was to determine the patients’ ability to be discharged from the unit directly to home; such patients were classified into the ‘Home discharge’ group and compared with those in the ‘Difficulty in discharge’ group. The degree of frailty was determined based on the Clinical Frailty Scale (CFS), and the relationship with the endpoint was analysed. A receiver operating characteristic (ROC) curve was created and the cut-off value was calculated with the possibility of home discharge as the state variable and CFS as the test variable. Logistic regression analysis was conducted with the possibility of home discharge as the dependent variable and CFS as the independent variable.

**Results:**

There were 75 patients in the Home discharge group and 63 in the Difficulty in discharge group. ROC analysis showed a CFS cut-off value of 6 or more, with a sensitivity of 70.7% and a specificity of 84.1%. The results of the logistic regression analysis showed a significant correlation between possibility of home discharge and CFS even after adjusting for covariates, with an odds ratio of 13.44.

**Conclusions:**

Based on the evaluation of the degree of frailty conducted in the COVID-19 treatment unit, it was possible to accurately predict whether a patient could be discharged directly to home after treatment CFS could be an effective screening tool to easily detect patients requiring ongoing hospitalisation even after the acute phase of treatment.

## Background

The spread of the novel coronavirus disease 2019 (COVID-19) has been explosive. As of 2023, it still has not been fully contained, and its effects has been prolonged in various countries throughout the world [1]. In Japan, the highly contagious Omicron variant became widespread from January 2022, and there have been repeated waves of infection, called the sixth, seventh, and eighth waves, where the number of infected individuals rapidly increased, followed by a subsequent decline [2]. Although this variant is more contagious than that seen at the beginning of the pandemic, the mortality rate among infected individuals is decreasing [3]. However, the risk of the infection becoming severe remains high among the older individuals with comorbidities [4, 5]. High risk patients and those with highly contagious symptoms are admitted to medical institutions to prevent the spread of infection and mitigate its severity.

The National Center for Geriatrics and Gerontology opened a dedicated ward for patients with COVID-19 (COVID-19 treatment unit), mainly admitting and treating older patients with comorbidities considered to have mild to moderate symptoms. In this unit, preparations for discharge were generally initiated from 10 days after onset of the symptoms. However, despite completion of the acute phase of the COVID-19 treatment, many patients could not be immediately discharged home due to a decline in their ability to conduct activities of daily living (ADL) caused by muscular or cardiopulmonary function disuse during treatment. The influencing factors for this phenomenon are thought to include advanced age and the existence of comorbidities [6, 7]. Furthermore, it is believed that the impact of hospitalization-related disuse syndrome may influence discharge to home [8, 9]. It has also been reported that frailty, not limited to COVID-19 patients, may have a negative impact on discharge destination and outcomes [10]. However, there are insufficient findings on the specific patient characteristics that tend to make discharge difficult. As a result of a preliminary analysis of data of patients admitted to the COVID-19 treatment unit in this centre from January to March 2022, we identified that there may be a correlation between frailty and home discharge [11]. However, there are few reports on the relationship between COVID-19 and frailty.

In this study, we formulated a hypothesis based on past findings, namely, evaluating the degree of frailty in patients positive for COVID-19 might help to predict if they could be discharged from the COVID-19 treatment unit directly to home after completion of the acute phase of treatment. If it is possible to predict cases that are highly likely to face challenges in being discharged home as early as the initial stage of COVID-19 treatment, providing early and targeted rehabilitation to improve their ADL abilities may be beneficial for preventing disuse during hospitalisation, thereby facilitating prompt discharge and a return to normal life after treatment [12, 13]. Therefore, this study aimed to investigate the relationship between the degree of frailty and home discharge of patients admitted to the COVID-19 treatment unit. This study will provide valuable implications for the future establishment of medical systems.

## Methods

### Participation

Of the 231 patients admitted to the COVID-19 treatment unit in this centre with positive COVID-19 polymerase chain reaction (PCR) test results during the 12-month period from January to December 2022, 138 (85 men; 53 women) were included in the analysis (excluding 16 patients younger than 65 years, 35 with hospital-acquired infections, 37 who were originally residents of nursing homes, and 5 with severe infections). In our center, we routinely conduct assessments of physical and cognitive function, including frailty, as part of our daily clinical practice. The assessment results are stored in the medical records, and the analysis was conducted retrospectively on the in-hospital medical records of the patients, ensuring anonymity. We provided an opt-out document to the patients, allowing them to ask questions about the research plan or refuse the use of their own data at any time [14].

### Assessment

The primary end-point was the patient’s ability to be discharged from the COVID-19 treatment unit directly to home. Therefore, the patients were classified into two groups: patients who were able to be discharged directly to home after completion of the acute phase of the COVID-19 treatment (‘Home discharge’ group) and patients who continued to stay in the hospital and required rehabilitation in order to be discharged home (‘Difficulty in discharge’ group).

The degree of frailty, as the main item for evaluating the correlation with the endpoint, was determined using the Japanese version of the Clinical Frailty Scale (CFS), translated by The Japan Geriatrics Society in 2021. CFS is a comprehensive index for evaluating the degree of frailty on a 9-point scale, as proposed by Rockwood et al. [15]. The scale allocates a high score for decline in both physical and cognitive functions as it takes into account an individual’s level of independence in ADLs and their need for nursing care. The evaluation does not require specialized equipment or a long period of time, making CFS is a simple index that enable comprehensive judgement based on clinical findings [16], This is advantageous in terms of ease of use, particularly in settings that require special infection control measures, such as COVID-19 treatment units.

Age, sex, body mass index (BMI), serum albumin level, severity of COVID-19, comorbidities, ADL (Functional Independent Measure; FIM), sarcopenia (calf circumference and grip strength), physical function (Short Physical Performance Battery; SPPB), and cognitive function (Mini Mental State Examination-Japanese; MMSE-J) were examined as secondary end-points.

The severity of COVID-19 was classified based on the Ministry of Health, Labour and Welfare criteria [17] as the following: mild (SpO_2_ ≥ 96% and no respiratory symptoms) moderate I (93%<SpO_2_ < 96% with dyspnoea or pneumonia), and moderate II (requiring oxygen therapy, with SpO_2_ < 93%). Patients with a calf circumference of < 34 cm for men and < 33 cm for women and a grip strength of < 28 kg for men and < 18 kg for women were determined to have ‘possible sarcopenia’ in accordance with the Asian Working Group for Sarcopenia (AWGS) 2019 criteria for sarcopenia [18]. SPPB is a simple but comprehensive physical function assessment battery that allocates a score of 0 to 12 by testing three items: 4 m walk, the chair stand test completed five times, and the standing balance test. The higher the score, the better the motor function [19]. MMSE-J is the Japanese version of the MMSE, a cognitive function test battery [20], which can be conducted relatively easily and allocates a score from 0 to 30; the higher the score, the better the cognitive function [21].

These evaluations were conducted approximately 5 days after onset of the symptoms, once it was confirmed that the fever had abated on the day when the in-ward rehabilitation started with the approval of the attending physician. All medical staff involved in the dedicated COVID-19 treatment unit complied with infection control measures in accordance with the instructions of the Infection Control Committee.

### Statistical analysis

The Student t-test, Mann-Whitney U-test, χ^2^-test, and Fisher’s exact test were used for comparison of the mean ± standard deviation and median (interquartile range] or percentage (%) descriptions for each end-point, and for comparison of the possibility of home discharge and each end-point. A receiver operating characteristic (ROC) curve was created with the possibility of home discharge set as the state variable and CFS set as the test variable, and the cut-off value was calculated at the maximum value of sensitivity, specificity, the area under the curve (AUC), and the Youden index. Furthermore, logistic regression analysis was conducted with the possibility of home discharge (Difficulty in discharge group = 1) set as the dependent variable and CFS (the binary variable at or above/below the cut-off value) as the independent variable. Spearman’s rank correlation coefficient was used to assess for multi-collinearity between covariates.

SPSS Ver. 28.0 (IBM, Armonk, NY, USA) was used for statistical analysis, and the level of significance was set at 1%.

## Results

**Fig. 1:**
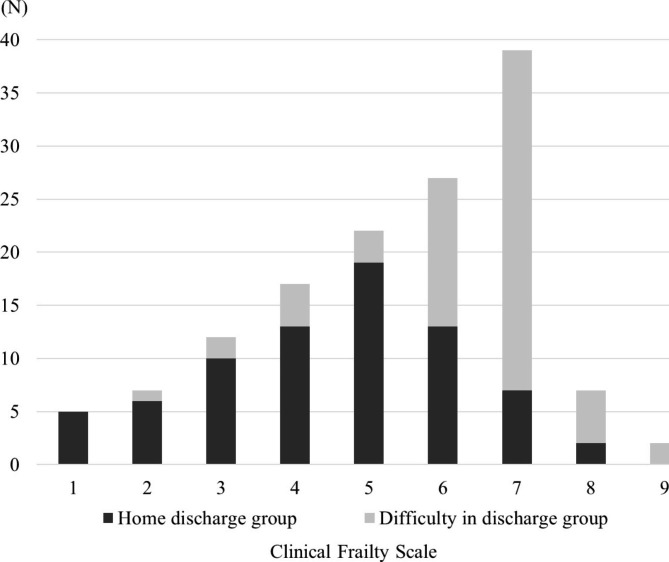
Distribution of Clinical Frailty Scale scores in study participants

There were no cases of infection among the medical staff in the dedicated COVID-19 treatment unit during the survey period, indicating that it was possible to safely provide medical care, nursing, and rehabilitation during the isolation period by adopting appropriate measures. The mean number of days spent in the ward by the 138 patients in the study was 11.2 ± 2.9 days, and the severity of COVID-19 at admission was mild for 58 patients, moderate I for 47, and moderate II for 32. There were 75 patients in the Home discharge group and 63 in the Difficulty in discharge group. The number of people who were able to be discharged home using the CFS score is shown in Fig. 1. The median (interquartile range) CFS score was 5 (3–6) in the Home discharge group and 7 (6–7) in the Difficulty in discharge group, and the number of cases that were difficult to discharge increased with the CFS score (P < 0.001, effect size=–0.55).

Table 1 shows the results of the comparison of each secondary endpoint based on the possibility of home discharge. While the data of enrolled patients were generally obtained without significant missing values, the evaluation of handgrip, MMSE-J, and possible sarcopenia was challenging for patients with severe dementia or impaired consciousness. Therefore, for these variables, we have reported calculated values excluding missing value. In the inter-group comparison, advanced age, female sex, low BMI, low serum albumin levels, low total FIM score, reduced calf circumference, low grip strength, possible sarcopenia, low SPPB score, and low MMSE-J score made discharging significantly more difficult. The relationship between comorbidities and the possibility of home discharge was only significant for dementia, and the severity of COVID-19 and the presence/absence of other comorbidities were not associated.

**Table 1:** Comparison of the clinical characteristics between the “Home discharge” group and the “Difficulty with discharge” group

	Overall (n = 138)	Home discharge group (n = 75)	Difficulty in discharge group (n = 63)	P value	Effect size
Age	82.7 ± 7.6	80.9 ± 6.8	85.0 ± 8.0	< 0.001	0.27
Sex_Female	53 (38%)	21 (28%)	32 (51%)	< 0.001	0.04
BMI (kg/m^2^)	21.4 ± 3.8	22.4 ± 3.7	20.2 ± 3.7	< 0.001	0.18
Albumin (g/dl)	3.1 ± 0.6	3.4 ± 0.5	2.9 ± 0.6	< 0.001	0.44
Severity of COVID-19- Mild - Moderate I - Moderate II	58 (42%) 47 (34%) 33 (24%)	33 (44%) 29 (39%) 13 (17%)	25 (40%) 18 (28%) 20 (32%)	0.208	0.11
Comorbidities (include duplicates)					
Cerebrovascular disease	30 (22%)	14 (19%)	16 (25%)	0.340	0.02
Respiratory disease	46 (33%)	26 (35%)	20 (32%)	0.717	0.01
Neuromuscular disease	18 (13%)	11 (15%)	7 (11%)	0.537	0.03
Dementia	81 (59%)	28 (37%)	53 (84%)	< 0.001	0.16
Hypertension	52 (38%)	25 (33%)	27 (43%)	0.250	0.02
Diabetes mellitus	27 (20%)	16 (21%)	11 (18%)	0.568	0.02
Osteoporosis	8 (6%)	5 (7%)	3 (5%)	0.727	0.03
Dyslipidemia	29 (21%)	17 (23%)	12 (19%)	0.603	0.01
Malignant neoplasm	22 (16%)	12 (16%)	10 (16%)	0.984	0.00
Heart disease	31 (23%)	17 (23%)	14 (22%)	0.950	0.00
Chronic renal failure	12 (9%)	7 (9%)	5 (8%)	1.000	0.01
Others	46 (33%)	23 (31%)	23 (37%)	0.468	0.01
FIM total	73 [31–98]	89 [73–108]	35 [22–66]	< 0.001	0.47
SPPB	4 [0–10]	8 [5-12]	0 [0–1]	< 0.001	0.52
MMSE-J *	20 [12-27]	25 [16-29]	12 [8-20]	< 0.001	0.44
Possible sarcopenia *	77 (63%)	32 (48%)	45 (82%)	< 0.001	0.12
Calf circumference (cm)	29.4 ± 3.9	30.4 ± 3.6	28.0 ± 4.1	< 0.001	0.30
Handgrip (kg) *	19.9 ± 9.8	23.7 ± 8.5	14.1 ± 8.9	< 0.001	0.48

Comparison of the clinical characteristics between the Home discharge group and the Difficulty in discharge group

Data are presented as the mean standard deviation and median [interquartile range]

MMSE-J: Mini-Mental state Examination-Japanese; SPPB: Short Physical Performance Battery

Student t test, Mann-Whitney U test, χ2-test, Fisher’s exact test. Effect size = Pearson’s correlation coefficient r or Cramer’s V

*MMSE-J, Handgrip and Possible sarcopenia determination excluded 16 patients due to missing values (8 persons in each group)

**Fig. 2:**
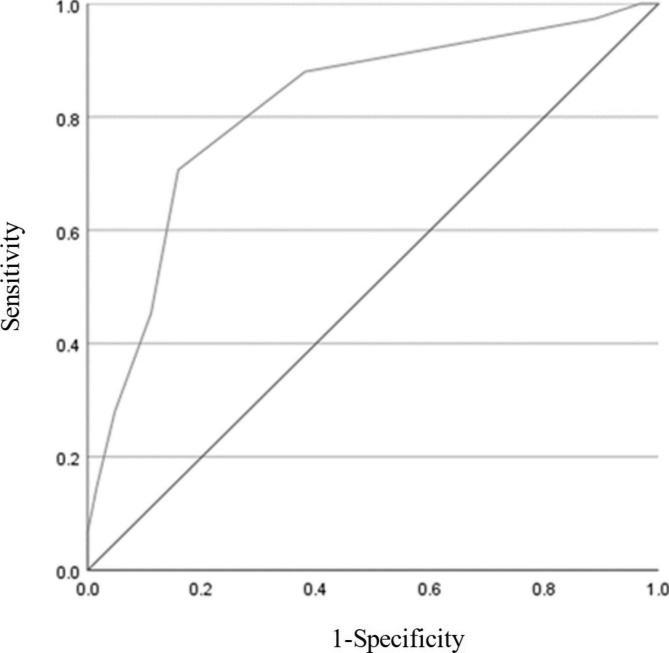
Receiver operating characteristic curves

Figure 2 shows the ROC curve with the possibility of home discharge set as the state variable and CFS set as the test variable. The CFS cut-off value was 6 or more, with a sensitivity of 70.7% and a specificity of 84.1%. The AUC was 0.816, with a ‘good’ prediction performance (Table 2).

**Table 2:** Sensitivity and specificity cut-off value for CFS

	CFS
Cut-off value	5 / 6
P	< 0.001
Sensitivity	71%
Specificity	84%
Maximum Youden index	0.55
Positive predictive value	74%
Negative predictive value	82%
AUC [95%CI]	0.82 [0.74–0.89]

CFS: Clinical Frailty Scale; AUC: Area Under the Curve; CI: Confidence Interval

Additionally, logistic regression analysis was conducted with the possibility of home discharge as the dependent variable, CFS (score ≥ 6: 1) as the independent variable, and age, sex, BMI, serum albumin level, and possibility of sarcopenia as covariates, as the latter items were found to have significant differences in the univariate analysis. Furthermore, significant inter-group differences were observed in terms of dementia and FIM, SPPB, and MMSE-J scores in the univariate analysis; therefore, these items were not included as covariates as they might have presented multi-collinearity with CFS (the Spearman’s rank correlation coefficients (ρ) were 0.72, − 0.87, − 0.85, and − 0.76, respectively). The results of this analysis are shown in Table 3. There was a significant correlation between the possibility of home discharge and CFS even after adjusting for covariates, with an odds ratio of 13.44 (95% confidence interval, 3.98–45.37), and the proportion of correct classifications was 81.6%.

**Table 3:** Relation between home discharge and CFS in the COVID-19 treatment unit

	Unadjusted			Adjusted	
	OR (95%CI)	P value		OR (95%CI)	P value
CFS (< 6 = 0)	Ref.			Ref.	
CFS (≥ 6 = 1)	18.48 (6.95–49.18)	< 0.001		13.44 (3.98–45.37)	< 0.001

Logistic regression analysis

CFS: Clinical Frailty Scale; OR: Odds Ratio; CI: Confidence Interval

Dependent variable: the Home discharge group (0) or the Difficulty in discharge group (1)

Independent variable: CFS < 6 (0) or ≥ 6 (1)

Covariate: Age, Sex, Body Mass Index, Albumin, Possible sarcopenia

Percentage of correct classifications: 81.6%

Ten patients could not be discharged home despite having a CFS score of less than 6. Almost all of these patients were older and had comorbidities that have been reported to increase the risk of exacerbation when infected with COVID-19, such as respiratory disease, diabetes, and cancer [22]. For such patients, the attending physician determined that continued hospitalisation was required for follow-up even after COVID-19 treatment had been completed. These patients were transferred to a normal ward for ongoing observation and rehabilitation. Conversely, there were 22 patients who were discharged home despite having CFS scores of 6 or higher. This included nine patients whose family members were well-equipped to care for them and 13 patients without excessive care burden placed on the family thanks to the use of home nursing care services, such as home-visit rehabilitation and day respite services; of note, these patients already required long-term care prior to their hospital admission due to COVID-19.

## Discussion

### Primary findings

The first noteworthy finding in this study was that CFS was shown to be an effective screening tool that can easily detect patients who require ongoing hospitalisation and rehabilitation intervention, even after the acute phase of treatment in a COVID-19 treatment unit. Moreover, this study demonstrated that it was possible to accurately predict whether a patient can be discharged directly to home based on the evaluation of the degree of frailty in the COVID-19 treatment unit.

### Usefulness of CFS assessment

The CFS assessment, which was used for the evaluation of frailty in this study, is a screening tool that can be used by anyone and that does not require any equipment, even in settings that require strict infection control measures, such as personal protective equipment. Rockwood et al., who developed CFS, also pointed out the usefulness of using it for patient triage under conditions that involve the spread of infection [15, 16]. SPPB and MMSE-J, which are used to evaluate physical and cognitive function, respectively, are similarly effective evaluation tools for screening and prognosis prediction [23, 24]. In this study, these indices also correlated with the possibility of home discharge; however, these evaluation tools require pre-training of evaluators and preparing dedicated measurement equipment for use in the infection control ward. On the other hand, CFS does not require any special training or equipment and can be determined in a short time-frame solely based on medical information, including a comprehensive evaluation of physical function, cognitive function, and ADL ability. Thus, CFS is considered superior to other evaluation methods, especially in COVID-19 treatment units.

In this study, the severity of COVID-19 was mild or moderate I in approximately 70% of the patients, and many of the patients were older people with a mean age of 82.7 years. Less than 20% of the patients were healthy without any frailty (CFS < 4), as related to both physical and cognitive function. Overall, patients admitted to COVID-19 treatment units during the spread of the Omicron variant were characteristically frail older people with various comorbidities. Generally, comorbidities are known to influence the feasibility of discharge [6, 7, 25]. However, the relationship between comorbidities and the possibility of home discharge was only significant for dementia, while no significant associations were observed for other comorbidities. This may be influenced by the relatively short duration of hospitalization in the dedicated COVID-19 treatment unit. An interesting finding in this study was that although the severity of severe acute respiratory syndrome coronavirus 2 (SARS-CoV-2) infection ranged from mild to moderate I, no correlation was found between the severity of infection and the possibility of home discharge. This finding is consistent with the results of previous studies that cognitive function and ADL impairment have a stronger correlation with prognosis than with the severity of SARS-CoV-2 infection in older patients (aged 80 years or older) [26]. Additionally, it was also considered that the impact of hospitalization-related disuse syndrome might affect the possibility of being discharged to home [8]. Furthermore, there have been reports suggesting an association between past falls, fear of falling, and both physical function and cognitive function [27, 28], it is possible that these factors may have indirectly influenced the progression of frailty. The results of this study demonstrated that in older patients with various comorbidities, it is important to consider not only to the severity of COVID-19 but also to the degree of frailty as well as the decline in ADL and cognitive function caused by the reduction in physical activity associated with hospitalisation.

### Prediction of discharge with CFS

As shown in the ROC analysis, the CFS cut-off value for predicting the possibility of home discharge directly from the COVID-19 treatment unit was 6, referring to moderate frailty. Furthermore, the degree of frailty had a stronger effect on the possibility of home discharge than that of age, comorbidities, or severity of COVID-19, and the odds ratio that the direct home discharge would be difficult was more than 13 times greater in patients with moderate or higher frailty compared to that in those without this level of frailty. It has been seen that older people who require even a small amount of help with ADL at home prior to admission, are prone to disuse syndrome during the acute phase of treatment and require ongoing hospitalisation after completion of treatment and rehabilitation intervention, even if the severity of COVID-19 is relatively mild [29, 30]. However, there were patients who could be discharged home even with moderate to severe frailty depending on environmental and social factors, such as having access to public nursing care services that started before the onset of COVID-19 or having family members who were well-equipped to care for them after their return.

This study was conducted during the spread of the Omicron variant, which is thought to be an attenuated virus strain that is more contagious than the Delta variant but with a reduced mortality rate [31]. However, the findings of this study demonstrated that when treating COVID-19 in older patients with moderate to severe frailty, the reduction in activity during the acute isolation period poses a risk of decline in ADL ability and onset of disuse syndrome, which are factors that impede their ability to return home. Therefore, it is important to implement appropriate physical rehabilitation and nutritional intervention [32] and consider therapeutic measures that aim to maintain or improve ADL from the initial stages of hospitalisation, thus promoting home discharge and social rehabilitation shortly after completing treatment.

For older people living in the community, practicing excessive self-restraint against going out and participation in activities reduces the opportunity for social interaction and increases the risk of reduced mental and physical function [33, 34]. The risk of a decline in ADL ability due to rest and reduced activity is particularly high in older people who are frail, require long-term care, and have decreased physical and cognitive function [35, 36], making targeted rehabilitation intervention essential from an early stage [6]. Most countries throughout the world have adopted a ‘living with COVID-19’ strategy, slowly returning to pre-COVID-19 life as much as possible. Indeed, the risk of developing severe illness from SARS-CoV-2 infection is certainly lower than that at the start of the pandemic in 2020. However, the findings of this study highlighted that when considering saving a person’s life as well as discharging them home to help them return to their own life, it should be borne in mind that the risks posed by infection are not necessarily low for frail older people with various comorbidities. The AWGS 2019 guidelines advocated for the importance of finding balance between preventing COVID-19 and maintaining function [37]. It is crucial to explore approaches that enable frail older people to live their lives while maintaining an equilibrium between infection control and activity, while also preventing the progression of frailty.

### Study limitations

This study had several limitations. First, this study was conducted within a limited period of time and at a single facility. Furthermore, the prognosis of potential future variants of SARS-CoV-2 remains unknown. Therefore, the results of this study cannot be generalised to all patients with COVID-19, and it is necessary to conduct further investigations in multiple facilities with different degrees of severity to determine if the observed trends are unique to patients with mild to moderate COVID-19 infected with the Omicron variant.

Next, this was a cross-sectional study that relied on evaluations conducted at a fixed timepoint, approximately five days after onset, once the fever had abated and the patient’s condition had stabilised. Therefore, this study cannot fully assess which has a stronger impact: the disuse syndrome caused by bed rest after hospitalization or the frailty that the patient had prior to hospitalization. However, the finding that the degree of frailty at initial evaluation had a significant effect on prognosis is very important; evaluating frailty is useful for predicting outcomes after completing the acute phase of treatment and for establishing appropriate measures. At present, it is necessary to follow up the progress of patients who had difficulty in being discharged directly to home and to clarify the effect of rehabilitation intervention and the long-term impact of COVID-19 on ADL in older people.

## Conclusion

This study investigated the characteristics of patients admitted to a COVID-19 treatment unit during the spread of the Omicron variant and explored the correlation between frailty and home discharge, as well as the prognostic value of frailty evaluation. The findings showed that CFS is useful as a screening tool to determine the need for continued hospitalisation after the acute phase of treatment, with a relatively high degree of sensitivity and specificity. In cases where patients have moderate to severe frailty, the conditions of isolation and reduced activity in a COVID-19 treatment unit should be considered factors that can hinder their return to home. It is important to consider measures aiming to maintain or improve ADL from an early stage of treatment, facilitating social rehabilitation shortly after completion of treatment.

## Data Availability

The data that support the findings of this study are available on request from the corresponding author. The data are not publicly available due to privacy or ethical restrictions.

## Abbreviations

COVID-19: Coronavirus disease 2019
ADL: Activities of Daily Living
CFS: Clinical Frailty Scale
BMI: Body mass index
FIM: Functional Independent Measure
SPPB: Short Physical Performance Battery
MMSE-J: Mini Mental State Examination-Japanese
ROC: Receiver Operating Characteristic
AUC: Area Under the Curve
CI: Confidence Interval
OR: Odds Ratio
